# Coexistence of Gonadal Dysgenesis and Mullerian Agenesis in a Female with 46,XX Karyotype: A Case Report

**DOI:** 10.31729/jnma.4287

**Published:** 2019-04-30

**Authors:** Santosh Kumar Jha, Rosina Manandhar, Veena Rani Shrivastava

**Affiliations:** 1 Department of Obstetrics and Gynaecology, Kathmandu Medical College and Teaching Hospital, Sinamangal, Kathmandu, Nepal

**Keywords:** *Gonadal dysgenesis*, *Mayer Rokitansky Kuster Hauser syndrome*, *Mullerian agenesis*, *primary*, *amenorrhea*, *46,XX*

## Abstract

Gonadal dysgenesis is a rare genetically heterogeneous disorder characterized by underdeveloped ovaries with consequent, impuberism, primary amenorrhea, and hypergonadotropic hypogonadism. Mullerian agenesis or Mayer Rokitansky Kuster Hauser syndrome is characterized by congenital aplasia of the uterus and the upper part (2/3) of the vagina in a woman with normal development of secondary sexual characteristics and a normal 46,XX karyotype. The association of gonadal dysgenesis and Mayer-Rokitansky-Kuster-Hauser syndrome is very rare and appears to be coincidental. We report a case of a 24 year old woman who presented with primary amenorrhea. The endocrine study revealed hypergonadotrophic hypogonadism. The karyotype was normal, 46,XX. Internal genitalia could not be identified on pelvic ultrasound and pelvic MRI. There were no other morphological malformations.

## INTRODUCTION

Gonadal dysgenesis with female phenotype is defined as a primary ovarian defect leading to premature ovarian failure as a result of failure of the gonads to develop or due to resistance to gonadotropin stimulation and occurs in <1 in 10,000 women.^1–3^

The Mayer-Rokitansky-Kuster-Hauser (MRKH) syndrome is a specific type of Mullerian duct malformation characterized by congenital absence or hypoplasia of uterus and upper two thirds of the vagina in both phenotypically and karyotypically normal females with functional ovaries and occurs in 1 in 5200 new born girls.^4–6^

An association between these two conditions appears to be coincidental.

## CASE REPORT

A 24 year old married female patient presented to Kathmandu medical college, department of Obstetrics and Gynecology 4 months back with complaints of primary amenorrhea, poor breast development and unable to conceive since last 6 months. There was no family history of consanguinity. Her growth and development were normal with normal intelligence. At the time of presentation, her height was 150 cm and weight 52 kg.

On examination, there was no facial dysmorphism, no features suggestive of Turner syndrome. No skeletal deformity was noted. Scoring of pubic was Tanner's Stage II and breast development were Tanner's Stage II (Figure 1A, 1B). No other affected members in the family were detected. Her sexual life was satisfactory. The abdominal ultrasound (Figure 2) findings were confirmed later by magnetic resonance imaging (MRI) of the abdomen and pelvis (Figure 3) which showed uterus and bilateral ovaries were not visualized with normal vagina (Figure 4) which end blindly superiorly and normal bilateral kidneys. Per speculum and bimanual pelvic examination revealed normal external genitalia, normal vagina which was blind superiorly, cervix was not visualized and uterus was non palpable.

Hormonal evaluation showed elevated follicle stimulating hormone (55.4 IU/L), Luteinizing hormone (11.4 IU/L), Estradiol (<5 pg/ml), Progesterone (0.1 ng/ml) and Thyroid stimulating hormone (1.46 mIU/L). Her karyotype (Figure 5) was 46,XX type.

Laparoscopy was adviced to the patient but she denied. We confirmed coexistence of two disorders namely, gonadal dysgenesis and MRKH syndrome in this patient. She is kept on estrogen (ethinyl Estradiol va-late) for breast development, bone health and on regular follow up.

**Figure 1A:**
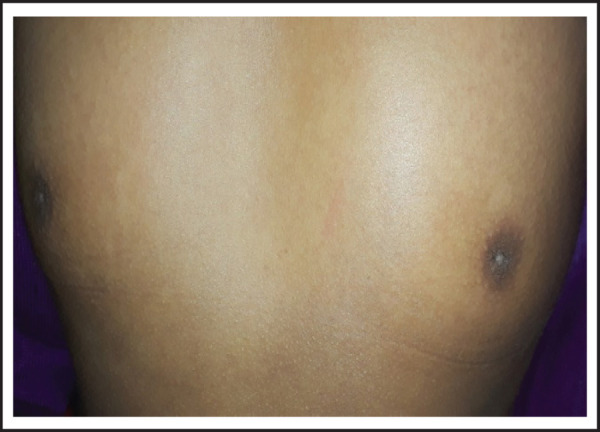
Breast Tanner's score II.

**Figure 1B:**
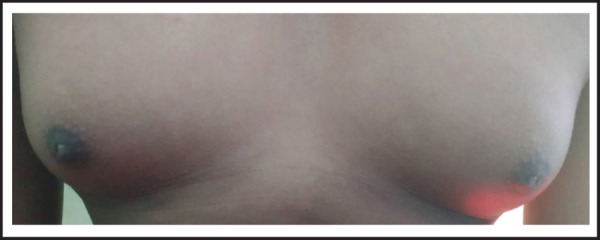
Improved Breast Tanner stage after receiving 3 months treatment.

**Figure 2.**
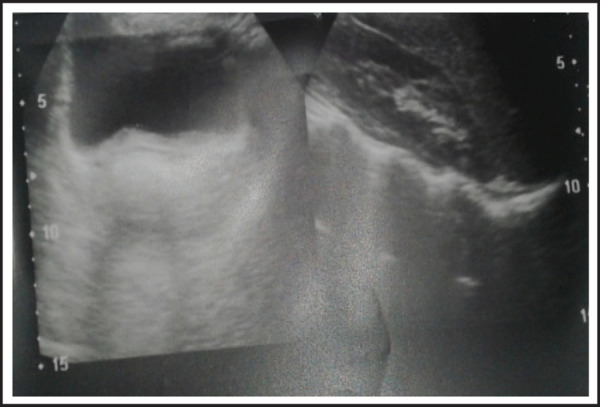
Ultrasound Examination- No Uterus identifieid behind the bladder.

**Figure 3.**
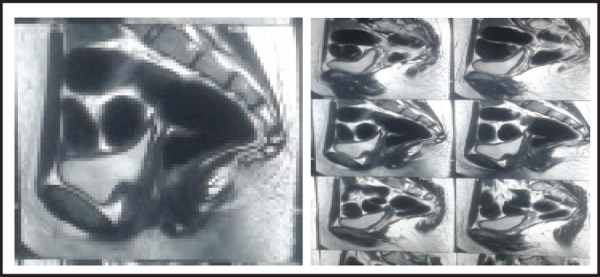
Magnetic resonance imaging examination sagital plane cut showing absence of uterus and ovaries.

**Figure 4.**
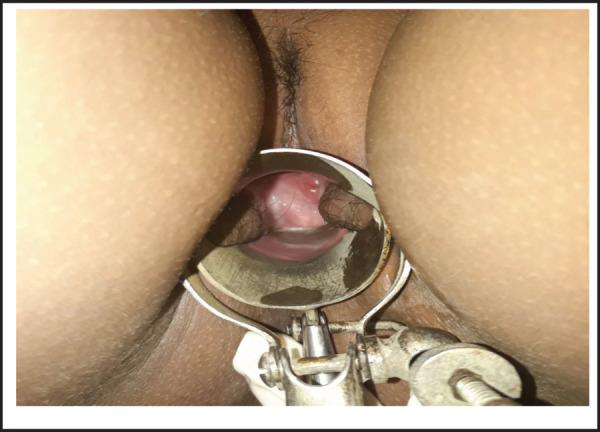
Normal vagina end blindly superiorly with dimpling.

**Figure 5.**
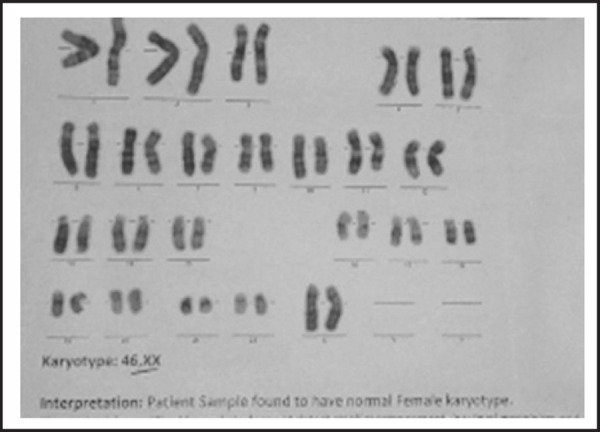
Karyotyping.

## DISCUSSION

Gonadal dysgenesis is the most common cause of primary amenorrhea and absent secondary sexual char-acteristics.^2^ Gonadal dysgenesis may arise from early defect in primordial follicle formation or defect in differentiation of the ovary.^4^ The molecular basis of this condition is still not known.^4^

MRKH syndrome is the second most common cause of primary amenorrhea, characterized by uterovaginal atresia in 46,XX female. Abnormalities of the genital tract may range from upper vaginal atresia to complete mullerian agenesis and may be associated urinary tract and/or skeletal abnormalities.^4^ It has been hypothesized to be due to abnormal activation of anti-mullerian hormone (AMH) expression or AMH receptor signaling in the female fetus, but no mutation in either AMH or AMH R have been reported.^4^ Mutations in WNT4 gene clearly involved in the mullerian duct genesis one of the important factor responsible for MRKH.^4^ Some investigators have differentiated two types of the syn-drome.^2,6^ The typical form (type A) is characterized by absence of both the vagina and uterus, leaving only symmetric uterine remnants, that is normal fallopian tubes and ovaries. The atypical form (type B) usually shows asymmetric or absent uterine remnants, hypo-plasia or aplasia of one or both fallopian tubes, and frequent urinary and skeletal congenital anomalies. The association of gonadal dysgenesis and MRKH syndrome is extremely rare.

Literature search revealed few such cases with 46,XX gonadal dysgenesis and MRKH syndrome, and the salient clinical features are discussed here for comparison. Levinson et al. described the first case of 46,XX go-nadal dysgenesis and MRKH syndrome in a 17-year-old female who presented with short stature, absence of vagina, secondary sexual characters, internal genitalia, and gonads.^3–5^ Bhandari et al. reported a case with short stature, impuberism, rudimentary vagina, and absent uterus and ovaries from Deheradun.^3^ Duta et al. reported a case of a 19-year-old female with short stature, normal secondary sexual characters, webbed neck, fusion of cervical vertebrae, scoliosis, atrial septal defect, right renal agenesis with malrotated lef kidney, and mul-lerian agenesis from New Delhi.^3^ Oyer et al. reported a case of neonate with 46,XX gonadal dysgenesis who presented with diaphragmatic hernia, doomed bicuspid aortic valve, and mullerian derivative defects.^3,4^ Kennerknecht et al. reported a 19-week-old fetus with 46,XX karyotype, normal female external genitalia, complete gonadal agenesis, large encephalocele, spina bifida, and omphalocele.^3^ All the studies mentioned above revealed that 46,XX gonadal dysgenesis with mullerian agenesis commonly presents with normal female phenotype with primary amenorrhea, impuberism, and hypergonado-tropic hypogonadism with or without somatic malformations, the later being extremely rare.

Unfortunately these two conditions compromise the prognosis of fertility of young patients. Hormone substitution therapy remains the only therapeutic option. It is aimed at triggering the development of secondary sexual characters and prevent osteoporosis. There remains the unsolved problem of infertility.

## Consent

**JNMA Case Report Consent Form** was signed by the patient and the original article is attached with the patient's chart.

## Conflict of Interest

**None.**
